# The complete chloroplast genome of *Torreya parvifolia*, a species with extremely small population in China

**DOI:** 10.1080/23802359.2020.1869611

**Published:** 2021-02-08

**Authors:** Lushui Zhang, Xingxing Mao, Xiyou Qian, Zefu Wang

**Affiliations:** a Ecological Security and Protection Key Laboratory of Sichuan Province, Mianyang Normal University, Mianyang, PR China; b Key Laboratory of Bio-Resource and Eco-Environment of Ministry of Education, College of Life Sciences, Sichuan University, Chengdu, PR China; c Heilongjiang Institute of Forestry Monitoring and Planning, Harbin, PR China

**Keywords:** *Torreya parvifolia*, chloroplast genome, phylogenetic analysis

## Abstract

*Torreya parvifolia* (*Torreya*, Taxaceae) is endemic in Sichuan, China. It consisted of an extremely small population with less than 100 wild individuals. In this study, the complete chloroplast genome of *T. parvifolia* was assembled using the Illumina data. The complete chloroplast genome of *T. parvifolia* is 137,106 bp in length. The genome consists of 119 genes in total, including 82 protein-coding genes (PCGs), 4 ribosomal RNA (rRNA) genes, and 33 transfer RNA (tRNA) genes. Phylogenetic analysis indicated that *T. parvifolia* was closely related to *T. fargesii*, *T. nucifera*, and *T. fargesii* var. *yunnanensis* with strong support.

*Torreya parvifolia* T.P. Yi, Lin Yang & T.L. Long, belonging to *Torreya* in Taxaceae family, is a species with extremely small population (Yi et al. 2006). It is reported that there are less than 100 individuals surviving in the wild (Pan et al. 2014). In this study, we reported the complete chloroplast genome of *T. parvifolia* for the first time and performed a phylogenetic analysis with 11 other species based on their complete chloroplast genomes.

We collected the fresh leaves of a wild *T. parvifolia* individual from Liangshan Yi Autonomous Prefectu, Sichuan Province, China (27°30′N, 102°56′E). Voucher specimen of the species was deposited in the Ecological Security and Protection Key Laboratory of Sichuan Province, China under the accession number: MNU-PHO-0160. The total DNA was extracted with the CTAB method (Doyle and Doyle 1987). We performed the whole-genome sequencing with HiSeq2500 Platform (Illumina, San Diego, CA) and obtained ∼10 Gb high-quality clean data. The complete chloroplast genome of *T. parvifolia* was *de novo* assembled with NOVOPlasty (Dierckxsens et al. 2017). The gene prediction was carried out by Plann (Huang and Cronk 2015) and Sequin (NCBI website). Finally, we obtained a chloroplast genome of *T. parvifolia*. The genome has been submitted to the GenBank under the accession number of NC_043866.1.

The complete chloroplast genome of *T. parvifolia* is 137,106 bp in length, with a GC content of 35.47% in total. The genome structure is similar to other Taxaceae species (Miu et al. 2018; Ge et al. 2019; Shin et al. 2019), with the loss of one copy of the inverted repeats (IRs). The chloroplast genome of *T. parvifolia* contains 119 genes, including 82 protein-coding genes (PCGs), 4 ribosomal RNA (rRNA) genes, and 33 transfer RNA (tRNA) genes.

To infer the phylogenetic position of *T. parvifolia*, we reconstructed a phylogenetic tree using the concatenated 64 PCGs sequences of *T. parvifolia* and 11 other species. The sequences of each gene were aligned by PRANK (Löytynoja 2014). RAxML (Stamatakis 2014) was performed to construct the phylogenetic relationships with 100 bootstrap replicates under the GTRGAMMA model. The maximum likelihood (ML) tree revealed *T. parvifolia* was closely related to *T. fargesii*, *T. nucifera*, and *T. fargesii* var. *yunnanensis* with strong support (Figure 1). Within the concatenated 64 PCGs sequences, *T. parvifolia* had 50, 187, and 162 varied sites with *T. fargesii*, *T. nucifera*, and *T. fargesii* var. *yunnanensis*, respectively. The genetic relationships of other *Torreya* species were identical with the previous study (Zhang et al. 2019) (Figure 1). Furthermore, we found *T. fargesii* did not cluster with *T. fargesii* var. *yunnanensis*. *T. grandis* and *T. grandis* var. *jiulongshanensis* were also not clustered together. It was possible because of the mis-identification of materials for sequencing, or the inaccurate published new varieties, which were only based on the characteristics of morphology in past years. In summary, the chloroplast genome of *T. parvifolia* could help us facilitate the identification and protection for *T. parvifolia*.

**Figure 1.**
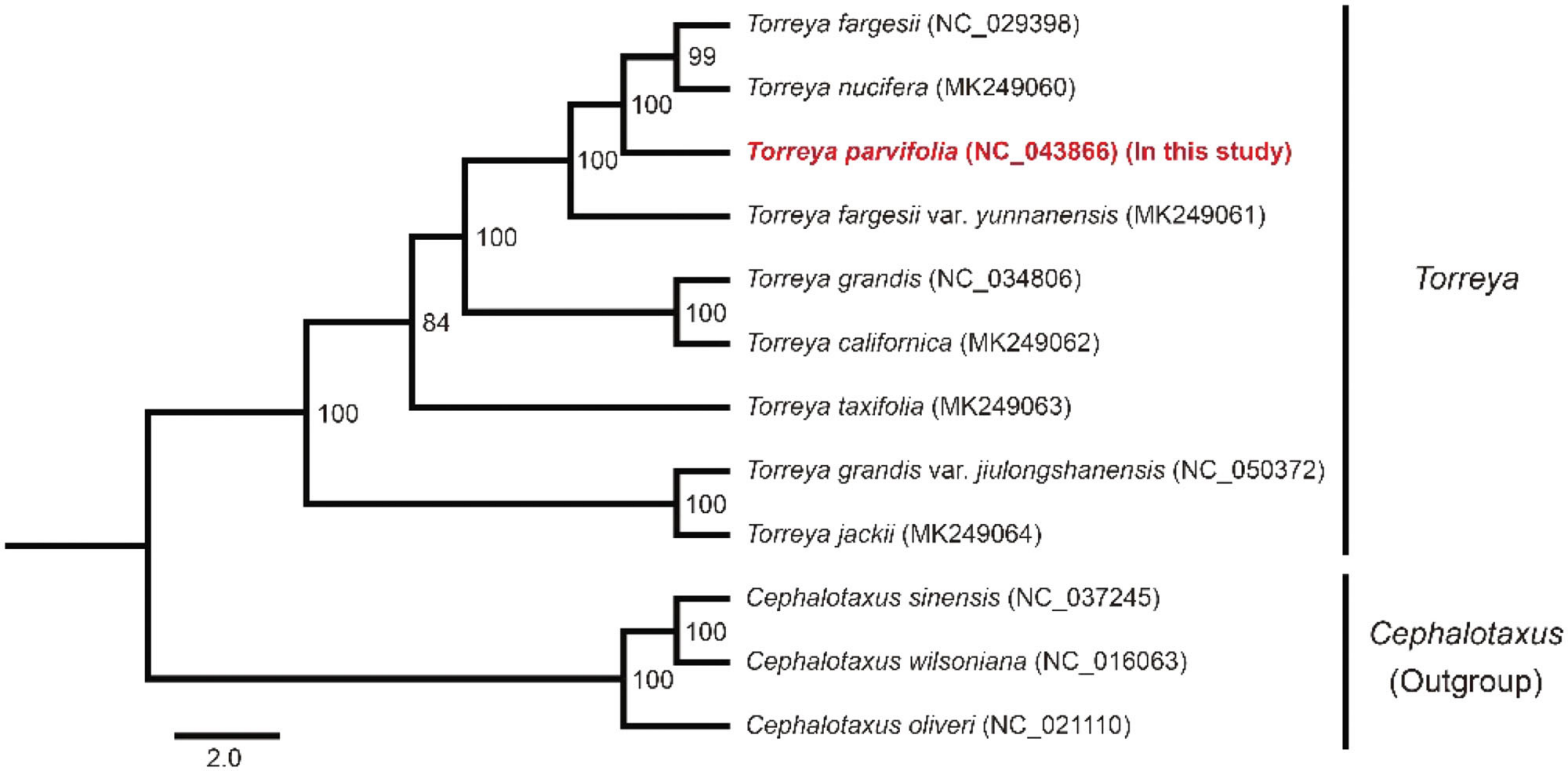
Maximum likelihood (ML) tree based on the concatenated data of 64 PCGs sequences of *Torreya parvifolia* and 11 other species. Numbers at the nodes indicate bootstrap values.

## Data Availability

The genome sequence data that support the findings of this study are openly available in GenBank of NCBI at (https://www.ncbi.nlm.nih.gov/) under the accession no. NC_043866.1. The associated BioProject, SRA, and Bio-Sample numbers are PRJNA683944, SRR13235763, and SAMN17048565, respectively. The voucher specimen of the species is free accessible at Ecological Security and Protection Key Laboratory of Sichuan Province, China with the no. MNU-PHO-0160.
